# Supplementary material for: The genomes of polyextremophilic cyanidiales contain 1% horizontally transferred genes with diverse adaptive functions
Source: eLife. 2019 May 31;8:e45017. doi: 10.7554/eLife.45017 (PMC6629376; doi:10.7554/eLife.45017)
Supplement: Transparent reporting form [file elife-45017-transrepform.docx]

***eLife’s* transparent reporting form**

We encourage authors to provide detailed information *within their submission* to facilitate the interpretation and replication of experiments. Authors can upload supporting documentation to indicate the use of appropriate reporting guidelines for health-related research (see [EQUATOR Network](http://www.equator-network.org/%20)), life science research (see the [BioSharing Information Resource](https://biosharing.org/)), or the [ARRIVE guidelines](http://www.plosbiology.org/article/info:doi/10.1371/journal.pbio.1000412) for reporting work involving animal research. Where applicable, authors should refer to any relevant reporting standards documents in this form.

If you have any questions, please consult our Journal Policies and/or contact us: [editorial@elifesciences.org](mailto:editorial@elifesciences.org).

**Sample-size estimation**

- You should state whether an appropriate sample size was computed when the study was being designed
- You should state the statistical method of sample size computation and any required assumptions
- If no explicit power analysis was used, you should describe how you decided what sample (replicate) size (number) to use

Please outline where this information can be found within the submission (e.g., sections or figure legends), or explain why this information doesn’t apply to your submission:

Not applicable to genome sequencing. Parameters for genome sequencing can be found in Table 1, and in the supplementary material, Figure 1S and Table 1S.

**Replicates**

- You should report how often each experiment was performed
- You should include a definition of biological versus technical replication
- The data obtained should be provided and sufficient information should be provided to indicate the number of independent biological and/or technical replicates
- If you encountered any outliers, you should describe how these were handled
- Criteria for exclusion/inclusion of data should be clearly stated
- High-throughput sequence data should be uploaded before submission, with a private link for reviewers provided (these are available from both GEO and ArrayExpress)

Please outline where this information can be found within the submission (e.g., sections or figure legends), or explain why this information doesn’t apply to your submission:

- Genomic, chloroplast and mitochondrial sequences of the 10 novel genomes, as well as gene models, ESTs, protein sequences, and gene annotations are available at <http://porphyra.rutgers.edu>.

- Raw PacBio RSII reads, and also the genomic, chloroplast and mitochondrial sequences, have been submitted to the NCBI and are retrievable via BioProject ID PRJNA512382.

-Criteria for HGT identification are described in the Materials and Methods section (“Detection of HGTs”)

**Statistical reporting**

- Statistical analysis methods should be described and justified
- Raw data should be presented in figures whenever informative to do so (typically when N per group is less than 10)
- For each experiment, you should identify the statistical tests used, exact values of N, definitions of center, methods of multiple test correction, and dispersion and precision measures (e.g., mean, median, SD, SEM, confidence intervals; and, for the major substantive results, a measure of effect size (e.g., Pearson's r, Cohen's d)
- Report exact p-values wherever possible alongside the summary statistics and 95% confidence intervals. These should be reported for all key questions and not only when the p-value is less than 0.05.

Please outline where this information can be found within the submission (e.g., sections or figure legends), or explain why this information doesn’t apply to your submission:

- The statistical approach for orthogroups and phylogenetic analysis is described in the Materials & Methods section (“Orthogroups and phylogenetic analysis”).

- When applied, statistical methods and their result can be found in the results section following a significance statement, e.g. “(GC-content of transcripts is normally distributed, Student's t-test, two-sided, p ≤ 0.05)”. Summary statistics information can be additionally found in the supplements. When applied, statistical methods and their result can be found in the results section e.g. “*All 11 Galdieria species showed significant differences in percent GC-content between native sequences and HGT candidates (GC-content of transcripts is normally distributed, Student's t-test, two-sided, p ≤ 0.05)*”, or “*This is the case for temperature-stressed G. sulphuraria 074W (categorical data, “native” vs. “HGT” and “differentially expressed” vs. “no differential expression”, Fisher’s exact test, p = 0)*.”. More precise summary statistics can be additionally found in the supplements and tables.

(For large datasets, or papers with a very large number of statistical tests, you may upload a single table file with tests, Ns, etc., with reference to sections in the manuscript.)

**Group allocation**

- Indicate how samples were allocated into experimental groups (in the case of clinical studies, please specify allocation to treatment method); if randomization was used, please also state if restricted randomization was applied
- Indicate if masking was used during group allocation, data collection and/or data analysis

Please outline where this information can be found within the submission (e.g., sections or figure legends), or explain why this information doesn’t apply to your submission:

Statistical group allocation was not applicable to the study design. The 10 novel genomes to sequence were chosen based on maximal molecular marker divergence within 36 available Cyanidiales strains at the HHU and ASU.

The Cyanidiales species were sorted into their respective lineage only after genome assembly and phylogenetic inference.

**Additional data files (“source data”)**

- We encourage you to upload relevant additional data files, such as numerical data that are represented as a graph in a figure, or as a summary table
- Where provided, these should be in the most useful format, and they can be uploaded as “Source data” files linked to a main figure or table
- Include model definition files including the full list of parameters used
- Include code used for data analysis (e.g., R, MatLab)
- Avoid stating that data files are “available upon request”

Please indicate the figures or tables for which source data files have been provided:
